# Parallel Distractor Rejection as a Binding Mechanism in Search

**DOI:** 10.3389/fpsyg.2012.00278

**Published:** 2012-08-09

**Authors:** Kevin Dent, Harriet A. Allen, Jason J. Braithwaite, Glyn W. Humphreys

**Affiliations:** ^1^Department of Psychology, University of EssexColchester, UK; ^2^School of Psychology, University of NottinghamNottingham, UK; ^3^School of Psychology, University of BirminghamBirmingham, UK; ^4^Department of Experimental Psychology, University of OxfordOxford, UK

**Keywords:** attention, feature binding, inhibition, visual search, conjunction search

## Abstract

The relatively common experimental visual search task of finding a red X amongst red O’s and green X’s (conjunction search) presents the visual system with a binding problem. Illusory conjunctions (ICs) of features across objects must be avoided and only features present in the same object bound together. Correct binding into unique objects by the visual system may be promoted, and ICs minimized, by inhibiting the locations of distractors possessing non-target features (e.g., Treisman and Sato, 1990). Such parallel rejection of interfering distractors leaves the target as the only item competing for selection; thus solving the binding problem. In the present article we explore the theoretical and empirical basis of this process of active distractor inhibition in search. Specific experiments that provide strong evidence for a process of active distractor inhibition in search are highlighted. In the final part of the article we consider how distractor inhibition, as defined here, may be realized at a neurophysiological level (Treisman and Sato, 1990).

## Analysis and Synthesis in Vision

Some of the most compelling cases in the neuropsychology of vision are patients who, following brain damage, experience selective loss of particular stimulus qualities. Patients with specific and restricted cortical damage may present with selective deficits for color (achromatopsia see, Zeki, 1990; Bouvier and Engel, 2006), motion (akinetopsia see, Zihl et al., 1983; McLeod et al., 1989; Zeki, 1991), or aspects of form processing (visual form agnosia, see, Riddoch and Humphreys, 1987; Goodale et al., 1991; Riddoch et al., 2008; Karnath et al., 2009). These neuropsychological cases provide convincing evidence that the different features of objects may be processed by brain systems that are at least quasi-independent.

Given that human vision has evolved such an analytic approach, the question naturally arises as to how the multiple features of different objects are properly combined (see Humphreys, 2001, for further discussion). Surely, such an analytic system should be prone to incorrect or illusory conjunctions (ICs) of features? Everyday experience of a seamless perceptual world may mislead us into thinking that “binding” these features of objects together is a trivial problem. However, the kinds of deficits that can occur following brain damage force us to reconsider. Patients suffering bilateral damage to parietal cortex may suffer severe deficits of perception, one aspect of which is frequent incorrect binding of features. Such patients presented with a red X and a green O may erroneously report having seen a red O – an IC (Friedman-Hill et al., 1995; see also Bernstein and Robertson, 1998; Humphreys et al., 2000). Even normal unimpaired observers asked to report relatively brief stimuli under conditions of attentional load may also report frequent ICs (Treisman and Schmidt, 1982). These ICs arise when the visual system is damaged and/or placed under attentional constraint, and are consistent with an early stage of analysis where the re-combination process is error-prone. The question thus arises as to the nature of the processes that prevent frequent ICs in healthy observers under everyday viewing conditions.

Here we consider some key models of selection and attention with particular emphasis on the mechanisms by which feature binding occurs and ICs are avoided. In particular we explore the role of inhibitory processes in the promotion of correct feature binding and the avoidance of ICs. This question of inhibitory processes was not fully explored by the earliest theories. For instance, one of the earliest and pioneering models of attentional selection was put forward by Broadbent (1957, 1958). Originally developed to account for data in the context of dichotic listening, the theory proposes that, following elementary feature analysis, further processing of information may be limited to stimuli possessing particular features. In Broadbent’s framework, a selective filter could be set which allows through only target features, but the fate of the rejected stimuli on this account is unclear. It is not considered whether rejected stimuli are equivalent to all items in the background and simply not subject to further processing, or whether rejected distractors can be inhibited below the background level.

Following our discussion of theoretical approaches below, we critically review some of the key experimental paradigms that have been used to address this question. Finally we suggest some possible neural mechanisms.

## Binding by Selection: Theoretical Approaches

**Figure 1:**
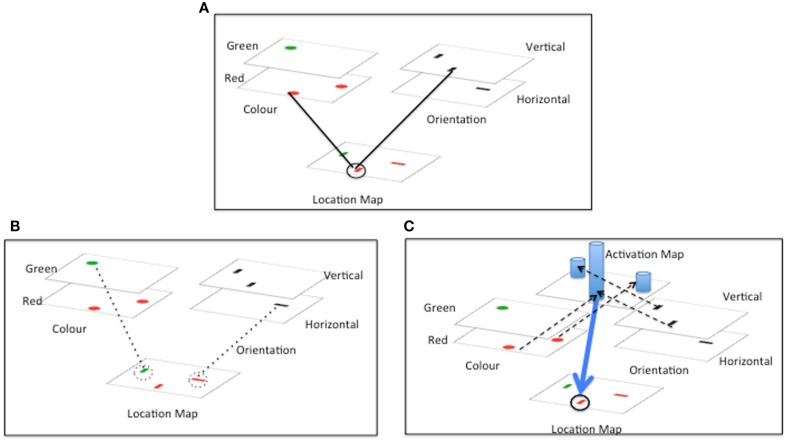
**Accounting for conjunction search**. Three influential accounts of conjunction search are depicted. In all cases search is for a vertical red bar, amongst vertical green and horizontal red bars. **(A)** Illustrates the basic location based cross referencing scheme that is the core of Feature integration Theory (FIT). **(B)** Illustrates the inhibitory revision of FIT proposed by Treisman and Sato (1990), and **(C)** illustrates the guided search revision proposed by Wolfe et al. (1989). **(A)** FIT: stimuli are decomposed into constituent features. Serial spatial selection by attention serves to recombine features based on location. **(B)** Feature inhibition revision of FIT: inhibition of distractors with non-target features (dotted lines) leaves target as only remaining uninhibited item. **(C)** Guided search revision of FIT: activation from target features is summed in an activation map. Spatial attention selects location with highest activation. Dotted lines indicate activation of activation map. Blue columns represent activation levels. Blue arrow represents direction of spatial selection by activation.

One important hypothesis concerning how features are bound is that spatial selection is key. Feature integration theory (FIT, Treisman and Gelade, 1980; Treisman, 1988; see Quinlan, 2003 for a review) proposes that, in order to properly bind together and represent combinations of features, spatial attention must select a particular location, and by doing so is able to cross-reference multiple features occurring at that location (see Figure 1A). Spatial selection of one location at a time can also solve the binding problem by highlighting only features at the attended location and minimizing the impact of other features, which are consequently not available for binding.

**Figure 2:**
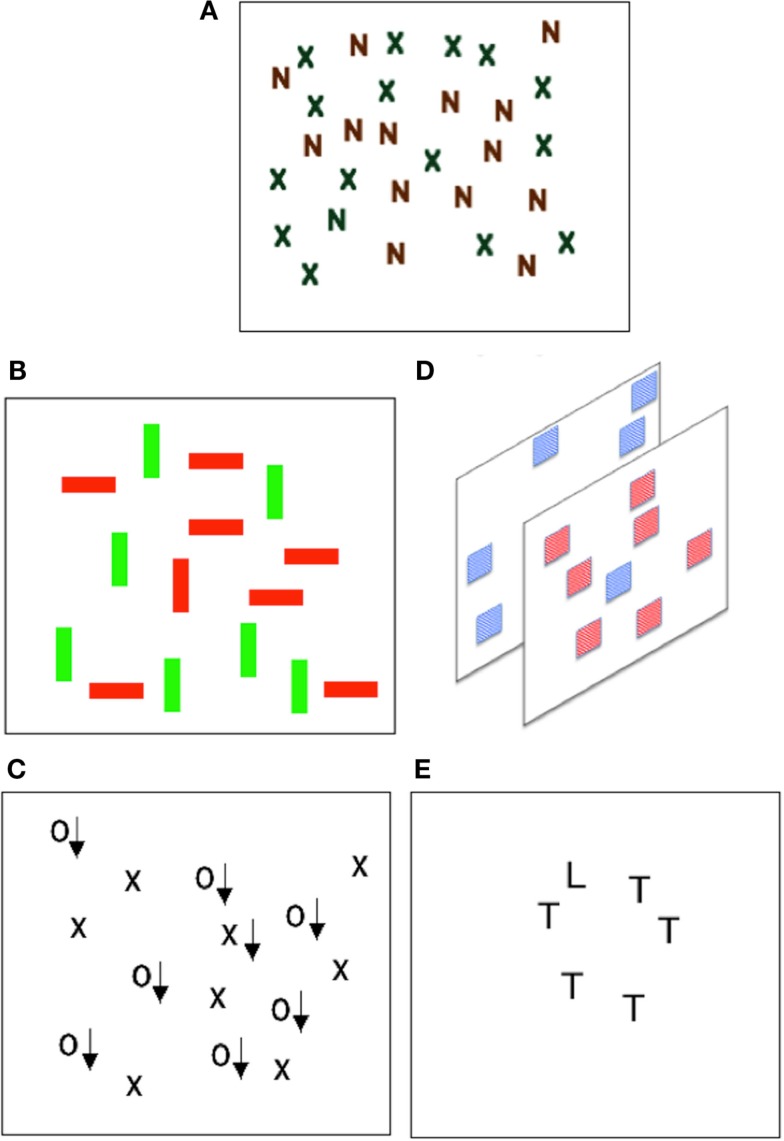
**Varieties of conjunction search**. Much of the research on selection and binding has used visual search tasks. Participants response times to find a particular target amongst distractors is compared. **(A)** Shows the classic “conjunction search” display, shown by Treisman and Gelade to produce inefficient search performance. **(B–E)** Each illustrate cases of efficient conjunction search. **(A)** Treisman and Gelade (1980). Inefficient color – form conjunctions. Finding the green N is difficult. **(B)** Wolfe et al. (1989). Efficient color – orientation conjunctions. Finding the red vertical bar is easy. **(C)** McLeod et al. (1988). Efficient motion – form conjunctions. Finding the moving X (arrows indicate motion) is easy. **(D)** Nakayama and Silverman (1986). Efficient color – depth conjunctions. Finding the front blue square is easy. **(E)** Duncan and Humphreys (1989). Efficient orientation – orientation conjunctions. Finding the L is easy.

However, research shows that sometimes feature binding can be achieved rapidly and without the serial selection of a set of individual locations required by FIT in it is unadulterated form. Several cases of efficient search (indicating little need for serial selection) for targets defined by conjunctions of features came to light in the late 1980s (see Figure 2 for illustration). Nakayama and Silverman (1986) showed that conjunctions of stereoscopic depth and either motion or color could be detected efficiently. Subsequently McLeod et al. (1988) demonstrated that conjunctions of movement and form could also be detected efficiently. Wolfe et al. (1989) returned to the case of color studied earlier by Treisman and Gelade (1980) and showed that conjunctions of color and orientation could be found efficiently given sufficiently large differences in the values of color and orientation used. Duncan and Humphreys (1989) also reported efficient search for targets distinguished from distractors only by the combination of form elements.

These findings of efficient conjunction search appear to challenge the fundamentals of FIT. Attentional Engagement Theory (AET, e.g., Duncan and Humphreys, 1989, 1992), on the other hand, proposes that feature and conjunction search do not necessarily differ in kind, but merely reflect different similarity relations amongst the stimulus elements. In AET search is directed by a template representing the target features; each item competes for selection with the outcome of this competition determined by the relative “attentional weight” assigned to each item. The attentional weight assigned to a stimulus is increased if it matches the template and decreased if it does not. Importantly, AET postulates that items that group together by sharing features can also change their attentional weights together (a process termed weight linkage). Weight linkage makes it easier for the system to reject groups of items in parallel. Thus in AET search difficulty is understood in terms of the roles of template matching and stimulus grouping enacted not just by positive excitatory changes but also by negative inhibitory changes.

Other authors have suggested ways in which FIT could be supplemented by additional guidance processes in order to account for efficient conjunction search. The Guided Search model (e.g., Wolfe et al., 1989, see Figure 1C) posits that search for a known target can be biased by top-down pre-activation of feature maps representing the expected properties of targets. Excitation from the feature maps is fed forward to a general activation map – where activation at each location is summed across the different incoming features. Values in the activation map dictate the probability that a particular location will be selected for further processing. Provided that the basic feature values are sufficiently discriminable, conjunction targets (with two preactivated features) will receive higher summed activity than distractors (with only one preactivated feature). The net result is efficient guidance of search to conjunction targets.

An issue with the Guided Search model is that the top-down modulation of feature maps will increase the activation of distractors that share features with the target. Increased distractor activation could lead to distractor features combining incorrectly with other activated features resulting in increased ICs (especially when the target itself is absent). Treisman and Sato, 1990, see also Treisman, 1988) suggested as an alternative a feature inhibition hypothesis in which those items with non-target features would be inhibited together (Figure 1B). This parallel inhibition by non-target features, like the suppressive weight linkage proposed by AET leads to reduced competition for selection from distractors, and efficient search. Importantly, selective inhibition by distractor features operates only when target and distractor features are so discriminable that distractor features can be suppressed without affecting target processing. Thus a significant benefit of an inhibitory as opposed to an excitatory bias (e.g., Guided Search), is that ICs are less likely to occur (since distractor features are inhibited). In this paper we seek to assess the feature inhibition hypothesis proposed by AET and suggested by Treisman and Sato (1990), evaluating whether the hypothesis meshes with what is known about visual attention and whether it provides a solution to the binding problem that is adopted by human vision.

There is a further key difference between FIT as proposed by Treisman and Sato (1990) and the AET as proposed by Duncan and Humphreys (1989). According to Treisman and Sato (1990) inhibition for each feature dimension is independent. That is, inhibition of stimuli based on color, would not take into account grouping by orientation. In contrast according to Duncan and Humphreys’s AET account, inhibition by different features is not independent, since negative losses of weight accrued to stimuli on the basis of one feature (e.g., color) may spread to other stimuli grouped with these items by other features (e.g., orientation). Under some conditions this can lead to stimuli that group on the basis of their conjunctive relationship (e.g., they differ from other groups only in how the same set of features combine) to be rejected together (see Found, 1998 for a demonstration).

In the next section we review the experimental approaches used to address whether there is suppressive rejection of distractors in general and also specifically in relation to the theories discussed above.

## Empirical Evidence: Attempts to Examine Distractor Suppression

There have been a number of approaches to testing whether target selection can take place by distractor suppression, and, if it can, how this process might operate. We briefly consider four types of study examining: (i) manipulations of distractor heterogeneity, (ii) trial-by-trial variations in stimulus properties, (iii) effects of stimulus foreknowledge, and (iv) probe-dot detection.

### Effects of distractor heterogeneity

Treisman and Sato (1990), Friedman-Hill and Wolfe (1995), McLeod et al. (1991), and Driver et al. (1992) all explored the relative importance of activation and inhibition in search by varying the number of features that characterized to-be-ignored or to-be-attended stimuli. The logic was simple. If participants actively deploy excitatory resources toward known target features, then increasing the number of possible target features should impede performance. In contrast, if participants deploy inhibitory resources toward known distractor features then the opposite should hold – in this case increasing the number of possible distractor features should impede performance.

Studies employing this method have generated data that support roles for both excitatory and inhibitory mechanisms in search. Treisman and Sato (1990) argued that, in color-form conjunction search, if distractors were added with features even further from the target than the existing distractors, then any mechanism tuned toward target features should be unaffected. In the standard conjunction case participants searched for a green 27° tilted bar amongst green 63° tilted bars and gray 27° tilted bars. The standard condition was compared against a condition where half of the distractors were replaced by green 90° and pink 27° tilted bars. If participants are positively tuned toward green and 27° then they should not be disrupted by the addition of pink and 90° features. The results showed that participants were in fact slower and less efficient when the number of distractor features increased, supporting the presence of inhibitory guidance away from non-targets.

Driver et al. (1992) also used this same logic in the context of search by motion direction. All the items were moving but participants selected items moving in one direction whilst ignoring items moving in another direction. The search items oscillated backward and forward along either a ±45° path. Additionally items moving along a particular path did so either coherently (all starting movement from the same point along the path) or incoherently (half of the items starting movement from each end of the path). If the target group moved incoherently there was no disruption to search, but if the non-target group moved incoherently search was disrupted. The greatest decrements to search occurred when both groups were incoherent. This pattern of results is consistent with a contribution from both excitatory and inhibitory processes but with an emphasis on the inhibitory.

On the other hand, similar studies have found no requirement for an inhibitory process. Friedman-Hill and Wolfe (1995) examined the case of selecting by color. Participants searched for an oddly oriented line amongst a color defined subset. Two critical conditions were compared: either participants had to select items of uniform color and reject two different color groups, or participants had to select two colors while rejecting a third possible color. Performance was much poorer when two colors had to be selected, supporting the involvement of excitatory processes that operate more efficiently when excitation can be tuned to a single target feature.

McLeod et al. (1991) asked participants either to select (a) moving and stationary items whilst rejecting items moving in a different direction, or (b) items moving in one direction whilst ignoring stationary items and items moving in different direction. The results clearly showed that the selection of stationary and moving items (condition a) was much more difficult than the selection of one moving direction (rejecting stationary distractors and distractors moving in a different direction, condition b). McLeod et al. interpreted this pattern of findings as evidence in favor of excitatory guidance of attention based on motion direction that may be disrupted by the inclusion of static items in the target group.

Clearly the evidence from studies manipulating distractor heterogeneity is not straightforward. It is difficult to unambiguously attribute changes in performance to attentional tuning (inhibitory or otherwise) in this task. As a consequence of manipulating the number of features present in the displays the grouping structure of the display also changes (see Duncan, 1995, for discussion); additionally the number of feature contrasts in the display also changes, and this may also alter bottom-up salience (see Julesz, 1986; Nothdurft, 2002). Ideally demonstrations of distractor inhibition should come from studies where display structure is held constant in the critical conditions.

### Trial-by-trial effects

Trial-by-trial priming effects have also been used to make arguments about inhibitory and excitatory guidance in search. Here the logic is that any inhibitory or excitatory effects will carry-over in time to the next trial. Koshino (2001) varied whether the target in a conjunction search on trial n had the same features as either the target or the distractors on trial *n* − 1. The results showed that, when the target was repeated, performance was speeded relative to when the target changed. In addition, there were disruptive effects when the target on the current trial took on the features of distractors on the previous trial (e.g., search for a Red X in Green X and Red N distractors, followed by search for a Green N in Green Vs and Magenta Ns), compared to when the features on the two trials were unrelated. Thus RTs were facilitated when features repeated and impaired when the target took the previous distractor features, consistent with a role for both excitatory and inhibitory processes.

Lamy et al. (2008) also examined trial-by-trial priming in the context of a color feature search. By using a color feature search task, in which participants searched for an odd colored item, they were able to independently manipulate whether the target and distractor features changed to new values, repeated the old values, or switched (previous target features becoming distractor features and vice versa). Relative to when the features changed to new values, there were both benefits of feature repetition and costs for feature switches. Thus performance was slower when the target appeared in the color of the previous distractors, and performance was slower again when the distractors additionally took on the previous target color. The authors interpret these particular effects as evidence for both target activation and distractor inhibition in search. Importantly, this distractor inhibition is reactive in the sense that it is set-up on-line based on whatever feature value the distractors happen to have. The inhibition does not take the form of pre-weighting a particular feature value (e.g., red) since the target and distractor feature values are not known in advance.

One interpretative issue with these studies of carry-over effects, is whether they directly reflect distractor inhibition, or rather the costs that might be involved when features from a past trial have to be re-bound to define the target (see Park and Kanwisher, 1994; Hommel, 2004). The necessity to re-bind the features may disrupt search even if there is no carry-over of the suppression of those features (from distractors onto targets). Therefore, ideally demonstrations of distractor inhibition in search should not rely on trial-by-trial effects alone.

### Effects of foreknowledge

Another method to try to assess the role(s) of inhibitory and excitatory guidance in search is to look at whether providing fore knowledge of either target or distractor features can lead to costs and benefits to search.

Shih and Sperling (1996) and Moore and Egeth (1998) both investigated the consequences of providing participants with foreknowledge regarding the likely color of an upcoming (feature-defined) target. Shih and Sperling (1996) showed that feature-based knowledge does not allow displays of a particular color to be completely filtered from vision. Participants viewed rapidly alternating displays of two different colors, with one of these displays containing the target. Increasing the probability that the target was a particular color did not modulate performance. Only when the target was a feature singleton in the displays did foreknowledge have an effect, consistent with a role for feature-based knowledge in spatial guidance of attention. Moore and Egeth (1998) used single displays composed of items of different colors, and manipulated the foreknowledge that participants had. The results showed that foreknowledge could change the speed with which targets could be found in time unlimited displays. However, when stimulus quality was degraded (e.g., with brief, masked displays) there was no effect of foreknowledge. The authors argue that had foreknowledge affected fundamental stimulus processing there should have been effects under these degraded conditions. Thus the authors suggest that rather than affecting fundamental feature processing, the effects of foreknowledge operate at a level of guiding attention.

In these tasks it is possible to observe both benefits for valid cues and costs for invalid cues, and at least logically these two effects are dissociable. Take the situation where the target appears 80% of the time in red and 20% of the time in green. Compared to the situation where target color is equiprobable, there can be benefits when the cue is valid, consistent with increased activation of items with likely features. There may also be costs on invalid trials, and these costs are at least consistent with inhibition of items with unlikely features. Relative to the case where the target was equally likely to be one of two possible colors, both Shih and Sperling, and Moore and Egeth reported benefits for validly cued color targets, and costs for trials where the initial target color cue was invalid (i.e., expect red and then the target was green). The presence of both benefits and costs is consistent with both excitatory and inhibitory processes. In particular Shih and Sperling (1996) say the following about participants who exhibit such costs: “they find the location of the odd item and, because it has the not-to-be-attended feature value, they suppress the information from that location – a true cost!… it is possible to enhance and also to suppress information from a single location.” (p. 773)

However, the results from manipulations of target foreknowledge are inconclusive. In particular, attentional selection may be understood as the outcome of a competition between different stimuli (e.g., Duncan and Humphreys, 1989). Thus, activating the properties of one stimulus (from an expectation of the target) may itself decrease the *relative* strength of a competing stimulus even though its absolute activation level remains constant. Thus the processing costs seen in these studies could be traced to increased competition from highly activated items in the cued color. In order to circumvent this problem what is needed is a measure of the *relative* loss of competitive strength for distractors that is independent of the search task.

As well as knowledge of the target, some studies have directly investigated the effects of providing knowledge of the distractors. Preview search, for example, presents one set of distractors for a preview period prior to adding a new set of items to the search display. Participants can use their foreknowledge of the distractors to influence their search performance. Under conditions where the preview is sufficiently long, the previewed distractors can have no impact on search – search progresses as efficiently in the preview condition as when only the second set of new items is presented (e.g., Watson and Humphreys, 1997). One striking result is that, if the new target carries features of the old, previewed items, then the target can be very difficult to detect – even when this target is a singleton in the new search display (Olivers and Humphreys, 2003; see also Braithwaite and Humphreys, 2003; Braithwaite et al., 2005 for similar results but with non-singleton displays). This last result is difficult to explain if there is only excitatory guidance of search to the new items with the old items forming a background that does not compete for selection, but it is consistent with the features of the previewed distractors being inhibited. Along with this, though, giving participants explicit foreknowledge of the likely color of the target helps to overcome the negative impact of carry-over from distractor features (Braithwaite and Humphreys, 2003). Again, the evidence is consistent with a role of both excitatory guidance of search to targets and inhibition of distractors.

One other point to note about these data from preview search is that they suggest inhibition not only of the features that will distinguish the previewed distractors from the new search items (e.g., their locations) but also of features carried by the distractors that are irrelevant to the search task. For example, in the studies of Braithwaite, Humphreys and colleagues (Braithwaite and Humphreys, 2003; Braithwaite et al., 2005), the target was defined by its identity and its color was irrelevant. Despite this, there was a negative effect on search when the target carried the distractor’s color. Interestingly, recent work has extended the range of conditions under which these effects occur beyond preview search. Thus when search is for a moving target and a group of static distractors are rejected, targets sharing color with the rejected static distractors are difficult to find (see Dent et al., 2011b). This evidence suggests inhibition of the rejected distractors as a group, and rejection of all the properties of the group, rather than just inhibition of the features that segment the target from distractors. This feature non-independence resonates more with the idea of weight linkage and spreading suppression within a distractor group (Duncan and Humphreys, 1989) than with the idea of feature-specific inhibition (Treisman and Sato, 1990).

Studies of target or distractor foreknowledge overall support the existence of both excitatory and inhibitory processes in search. Given that any effects will be due to both the properties of both perceptual processes and to any limitations or strengths of the foreknowledge system, it is important to find converging evidence from other methods as well.

### Probe-dot studies

The efficiency of processing a probe dot presented at different locations in a display has been used as a tool to explore the allocation of attention in search. The probe-dot task was first coupled with search in order to explore the related phenomenon of inhibition of return (IOR). IOR refers to the reduction in processing efficiency that follows the withdrawal of spatial attention from a previously attended location (see Posner and Cohen, 1984, for the initial demonstration and Klein, 2000 for a review). Klein (1988) suggested that IOR could be an important mechanism in search allowing examined items to be marked as rejected, acting as a “foraging facilitator,” promoting sampling of new unprocessed stimuli. In order to investigate this link, Klein (1988) had participants carry out a search task and then presented a probe either at the previous location of a distractor or at a previously unoccupied location in the background. He found that probes falling at the locations of earlier distractors were more difficult to detect than probes falling on prior background locations. This difference was increased when the search task was difficult (e.g., involving serial scanning of attention) compared with when it was easy (in feature search tasks) – which is important because it shows that the effect can not be due to masking from an earlier item at the same location. Subsequently Müller and Von Mühlenen (2000) and Takeda and Yagi (2000) have shown that these costs for the detection of probes on distractors is stronger again if the search items remain visible when the probe appears. These studies are consistent with the view that, in difficult serial search, distractors can be inhibited below the activation levels associated with the background as a result of IOR.

Whilst important in supporting the general principle of inhibitory processes, the notion of IOR, however, is different from the idea of spreading suppression or the parallel inhibition of distractors with a shared feature. By definition, IOR is applied serially across a display and only to items that have been selected and rejected. In contrast, spreading suppression and feature-based distractor inhibition are proposed to operate during selection – reducing the impact of distractors on target selection and on the chances of their features binding with those of targets. Illustrating this difference, Olivers et al. (2002) tested preview search under conditions where participants had to serially search the previewed distractors prior to searching the new stimuli, which should maximize IOR of the old stimuli. They found people were less, not more, likely to exclude previewed distractors under these conditions and concluded that the rejection of a common set of distractors in preview search took place using mechanisms distinct from IOR.

**Figure 3:**
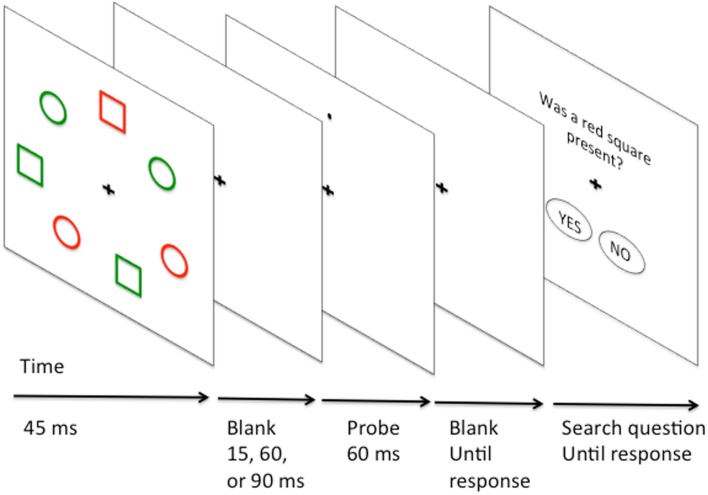
**Illustration of Kim and Cave (1995) probe-dot study of conjunction search**. Target is a red square. Distractors share either shape (green square), color (red circle) or neither feature (green circle) with the target.

The earlier study of Klein (1988) examined broadly how search difficulty affected subsequent probe detection. However, Klein (1988) and related studies of IOR did not examine tasks where there was an opportunity for feature-based guidance of attention. In these earlier studies the relative excitation or inhibition of distractors possessing different features within a display was not addressed. To address the question of the relative excitation and inhibition of different types of distractors during selection, Kim and Cave (1995) examined probe-dot detection in the context of search for conjunctions of color and form (e.g., find a red square amongst green squares, red circles, and green circles, see Figure 3 for illustration). Following conjunction search participants responded to the presence of a probe dot (present 25% of the time). The probe dot could appear on the target, or on a distractor. In general RTs were fastest for probes on the target, slower for probes on distractors that shared either color or form, and slowest of all on distractors that shared neither feature with the target. Unfortunately, probes were never presented on a neutral blank background location, and so it is difficult to judge whether the pattern of results should be attributed to target feature activation, or distractor inhibition.

A subsequent study by Cepeda et al. (1998)addressed the possibility of distractor inhibition by including a neutral baseline condition. Participants searched for a color singleton target and probes were presented either on distractors or in the background. The authors included a structured grid in the background and the elements making up the search items were also made up of the grid – so a probe fell equally far from a grid/distractor contour in the control and experimental conditions, controlling for masking. The results showed suppression of probe detection at distractor locations, even when masking was controlled. Additionally, Cepeda et al. compared the performance of two groups of participants: the active search group searched for the target and then detected the probe, whereas the passive group only detected the probe after viewing the same displays passively. Bottom-up masking is equated for the two groups, thus any effect in the active group, must stem from top-down attentional modulation. The results revealed distractor suppression that was specific to the active group and did not occur for the passive group, consistent with a role for top-down inhibition but only when needed for selection. Müller et al. (2007) reported similar results in the context of an efficient search for an orientation singleton. Müller et al. (2007) also tackled the issue of masking by comparing an active and passive group of participants. The results showed that probes presented on distractors were detected more slowly than probes presented on the background, and this effect was much greater in the active group, supporting a role for top-down inhibition.

Humphreys et al. (2004) applied probe-dot detection to the preview search task, controlling for masking in the manner introduced by Cepeda et al. (1998). They showed that probes presented on old items were more difficult to detect than those presented on the background. Interestingly in their conjunction search task no effects were seen. However it should be noted that the effects in the preview search based on accuracy were small and it may be that effects in the conjunction search were missed. Allen and Humphreys (2007) measured the ability to detect a contrast increment probe on previewed items in a psychophysical paradigm. By measuring the minimum increment that could be reliably be detected on the previewed items, they were able to show that previewed items are effectively reduced in contrast in the visual system. These studies suggest that distractor suppression may be particularly strong under conditions where one irrelevant set of items can be filtered over time.

**Figure 4:**
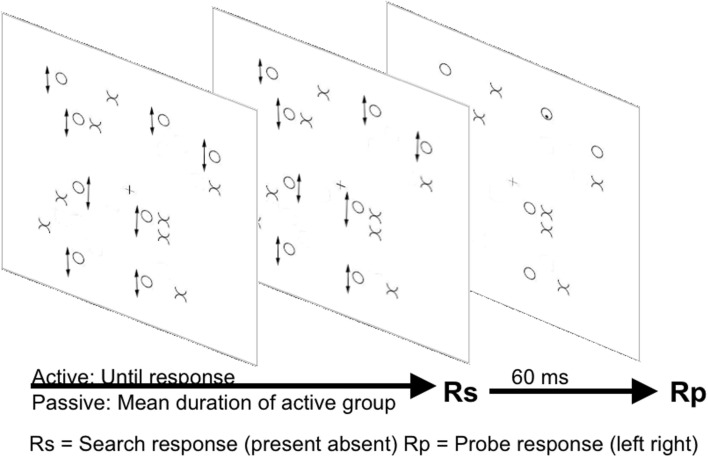
**Illustration of the Dent et al. (2012) paradigm**. Arrows next to items indicate oscillatory motion. Arrows underneath stimuli panels indicate the passage of time.

Recent research from our lab (Dent et al., 2012, see Figure 4) has investigated distractor suppression in a further efficient search task, in this case involving target conjunctions defined by movement and form (e.g., McLeod et al., 1988). As described earlier there is disagreement in the literature regarding the mechanisms underlying efficient search for conjunctions of movement and form. Studies manipulating distractor heterogeneity have drawn conflicting conclusions. McLeod et al. (1991) suggested a preeminent role for activation of moving items, whilst Driver et al. (1992) suggested that distractor inhibition was the more important mechanism. Dent et al. (2012) used the probe-dot task in an attempt to resolve this issue. Dent et al. (2012) showed that, when participants searched for a moving X amongst moving O and static X distractors, there were costs for probes presented on static X distractors consistent with inhibition of the location of the static items. The inhibition in conjunction search was much larger than any inhibition in any feature search condition. Furthermore this inhibition applied only to a group of participants actively engaged in the search task, and not to a group who viewed the same search displays passively responding only to the probe. Interestingly we found that when the displays were viewed passively responses were in fact slower to probes presented on moving compared to static items. This static advantage changed into a disadvantage when participants were actively engaged in search. This slowing depending on the participants being actively engaged in search argues against any enhanced masking effect being responsible for probe inhibition under active search conditions.

Results from probe-dot tasks provides strong evidence for inhibition in search. We note that probe-dot studies have significant advantages, for measuring attentional modulation in search, over the other methodological approaches reviewed above. By comparing probe detection at distractor and at blank locations the experiments include an appropriate neutral baseline missing from studies manipulating foreknowledge and target probability. Inclusion of an appropriate neutral baseline is crucial for assessing the polarity of attentional guidance (excitatory or inhibitory) in search.

As noted above, most attempts to examine excitatory and inhibitory contributions to search have manipulated the nature of the search task itself. Here, the experimental observation changes the very nature of the task under scrutiny. The method for measuring inhibition is an integral part of the search task, and is measured with some response to the search display. By changing the nature of the search task the experimenter may also inadvertently change display-wide grouping or bottom-up salience. We argue that the probe-dot task provides a tool that allows the experimenter to measure components of search, without disrupting or changing the nature of the search task under scrutiny. Since the probe task is a tool that is independent of search *per se*, probe detection can be examined as a function of search activity. In other approaches where the search response itself provides the index of attentional priority it becomes difficult to vary the degree of engagement with the search task without also varying the nature of the search stimuli. Additionally, probe detection provides a quite direct and relatively unambiguous measure of attentional priority that by-passes issues related to rebinding of features common to trial-by-trial approaches to measuring distractor activation and inhibition.

In addition to providing evidence for the existence of feature inhibition in search, results from the probe-dot task provide important constraints on the nature of this inhibition. Note that in this task the probe does not necessarily share any features with the distractor on which it is presented apart from location. Thus the probe itself would not be represented in the same feature maps as the search distractors. Thus any disadvantage for probe processing is unlikely to be rooted in inhibition in the feature maps themselves but rather from the coding of priority at the level of a general masters alience map.

## Conclusion

Our review so far indicates that there is evidence for the parallel suppression of distractors under conditions in which targets can be efficiently segmented from distractors, with the inhibition across groups of distractors with a common feature not shared by the target. This result is consistent with ideas put forward by both Treisman and Sato (1990), in their modification of FIT, and Duncan and Humphreys (1989), in their AET. The data additionally suggest that the suppression is found not only for the features distinguishing targets from distractors but also for irrelevant features carried by the rejected distractors. This is consistent with groups of distractors being rejected together (Duncan and Humphreys, 1989) rather than there being suppression of particular feature maps (Treisman and Sato, 1990). On top of this there is evidence that positive expectancies for target features can also bias search, to at least some degree offsetting the influence of spreading inhibition. We conclude that efficient selection of targets, particularly where their features could bind incorrectly with the features of distractors, is based on dual mechanisms of distractor suppression and excitatory guidance of attention to targets (see Braithwaite and Humphreys, 2003, for articulation of a dual attention set hypothesis).

## Neural Mechanisms

MacLeod et al. (2003), MacLeod (2007) cautions against the identification of inhibition at the cognitive level with neural inhibition. We endorse this cautious approach as the relationship between cognitive level inhibition and neurophysiology is a complex one and almost certainly a whole network of brain areas will be involved in distractor suppression in search. It is also not necessarily the case that decreased neural activation corresponds to functional inhibition at a cognitive level. For example an fMRI study by Allen et al. (2008) showed that, in preview search when people were ignoring faces and selecting houses or vice versa, activation in stimulus specific processing areas sensitive to the ignored stimuli *increased* rather than decreased (the fusiform face area FFA for faces and parahippocampal place area PPA for houses). Thus when particular features are used to signal inhibition, increases in activation in mid-level feature-specific areas may occur. This might reflect direction of an inhibitory signal itself or the initial allocation of attention to the distractor in order to subsequently inhibit it (see Tsal and Makovski, 2006).

Other recent research using fMRI (e.g., Dent et al., 2011a; Payne and Allen, 2011) has shown that increased efficiency in preview search can be related to decreased activation in V1 when the final search display is present. Similar results have been found when participants know that a target will not appear in a particular location (Serences et al., 2004; Sylvester et al., 2008). One hypothesis might be therefore that location specific feature inhibition could manifest as reduced activation in V1, although this remains to be demonstrated more generally when participants select targets by features rather than using temporal signals (as in preview search). Specifically, in the context of feature inhibition, one may question the suitability of V1 deactivation as a causal mechanism. Reduced activation in V1 may be taken to imply reduced elementary perceptual processing at specific locations or activation of a reduced number of locations. At both a theoretical level (e.g., Wolfe and Horowitz, 2004) as well as an empirical level (e.g., Shih and Sperling, 1996; Moore and Egeth, 1998) there are reasons to prefer the idea that feature inhibition does not completely suppress pre-attentive stimulus processing. Wolfe and Horowitz (2004) point out that if directing attention away from a particular feature results in reduced elementary processing of that feature, then (i) the very basis for the guidance will be undermined over time as the guiding feature is degraded, and (ii) if fundamental stimulus processing is affected then it may be difficult to rapidly reconfigure the system to accomplish certain tasks, for example deciding if a green object has a red spot. Certainly it remains to be seen if such V1 deactivation is a general phenomenon that can be driven by a range of features or whether it is specific to spatio-temporal cueing.

One way to reconcile the idea that feature inhibition is realized by reductions in V1 activation is to view V1 activity, as revealed by the BOLD signal, as reflecting a salience representation (e.g., Li, 2002), rather than stimulus processing efficiency *per se*. It is certainly possible that the master-map of spatial locations as described by Treisman could be housed in V1, further research is needed to address this question. Clearly other structures including parietal and frontal areas are also involved in directing the deactivation of V1. One possibility is that these areas may code a spatial representation of the to-be-ignored distractors (see Allen et al., 2008) and that this template, in conjunction with frontal areas, can be used to direct changes in the response of V1 neurons. Thus one potential circuit to implement feature inhibition would be that *increased* activation in feature-specific areas (V4, MT, IT, PPA, FFA) signals the to-be ignored feature, and setting of appropriate inhibitory weights to translate feature activation into reduced priority at the master-map level. Subsequently, interactions between feature-specific regions and downstream areas in the parietal cortex and precuneus create a spatial template that is used to coordinate location specific deactivation in V1. There are also other candidate structures that could implement a master salience map. Notably, the temporo-parietal junction has been highlighted as an important neural structure for bottom-up attentional capture (see Corbetta and Shulman, 2002), and recent computational modeling work suggests important links between salience as implemented in a neuro-computational model and TPJ activity observed in an fMRI study of preview search (Mavritsaki et al., 2010).

## Summary and Conclusions

Feature inhibition has been hypothesized to play an important role in search, guiding attention away from distractors and preventing ICs. Here we show by exploring the literature, that there is a theoretical niche for such a mechanism. Probe-dot studies are highlighted as being well suited to providing behavioral evidence for this mechanism. Although there is good evidence for inhibitory guidance of attention, there is also good evidence supporting the proposal that the multiple features of objects may not always be independent targets for inhibition, inhibition of one feature of an object may inadvertently lead to the inhibition of other features of the same object. Such feature non-independence of suppressive mechanisms in search is consistent with the AET account (Duncan and Humphreys, 1989), but problematic for guided search models (e.g., Treisman and Sato, 1990; Wolfe et al., 1989) that are rooted in the FIT tradition (Treisman and Gelade, 1980). We suggest that both positive excitatory and negative feature inhibition are necessary to permit efficient selection. We speculate that a brain network involving feature-specific areas V4, MT, FFA, PPA, feature general spatial representations in parietal and precuneus areas, top-down control structures in frontal cortex, and a early sensory regions, e.g., V1 work together to implement feature inhibition.

## Conflict of Interest Statement

The authors declare that the research was conducted in the absence of any commercial or financial relationships that could be construed as a potential conflict of interest.
